# Effect of led photobiomodulation on tooth movement, gingival hypertrophy and pain in response to treatment with fixed orthodontic appliance

**DOI:** 10.1007/s10103-025-04444-5

**Published:** 2025-04-18

**Authors:** Anja Sedej, Nika Svetina, Aljaz Golez, Ksenija Cankar, Helena Ban Frangez, Igor Frangez, Maja Ovsenik, Lidija Nemeth

**Affiliations:** 1 Orthos Institute, Ljubljana, Slovenia; 2 Public Health Center Radovljica, Radovljica, Slovenia; 3 University of Ljubljana, Ljubljana, Slovenia; 4 Ljubljana University Medical Centre, Ljubljana, Slovenia

**Keywords:** Photobiomodulation, Orthodontic tooth movement, Gingival hypertrophy, Pain

## Abstract

Photobiomodulation (PBM) is a form of treatment that uses low-power red and near-infrared light to stimulate tissue repair and regeneration at the cellular level. 32 subjects (198 teeth examined), 10 males and 22 females aged 14.6 ± 2.0 years, with mild dental crowding were included in a randomised, controlled clinical trial. The patients were treated with a fixed orthodontic appliance (FOA). Subjects were randomised into an experimental group (a PBM group irradiated with an LED light source with wavelengths of 625 nm, 660 nm and 850 nm simultaneously and an irradiance of 16 mW/cm²) and a placebo control group that received non-therapeutic irradiation with visible light. PBM therapy began within the first 2 days of appliance insertion and was administered twice weekly for 4 weeks. The rate of tooth movement (a change in distance at the same selected point on the occlusal plane of the tooth determined by measurements on 3D models), the presence of gingival hypertrophy (with a free gingival margin of at least 1 mm occlusal to the enamel-cement junction), the plaque index (PI), the sulcus bleeding index (SBI) and the subjective pain sensation using the visual analogue scale (VAS) were monitored. In the experimental PBM group (*N* = 14), the rate of movement with the FOA was statistically significantly higher at both 1 week of placement (0.5 mm [95%CI: 0.4–0.8]) and 4 weeks (1.1 mm [95%CI: 0.8–1.4]) than in the placebo group (*N* = 18), where the values were (0.4 mm [95%CI: 0.2–0.5]) at 1 week and (0.6 mm [95%CI: 0.4–0.9]) at 4 weeks. A lower incidence of gingival hypertrophy was observed in the PBM group (21.4%) than in the placebo group (55.6%) after 4 weeks (Mann-Whitney U-test, *p* < 0.05). PBM with LED accelerated orthodontic tooth movement during the levelling and alignment phase and reduced the incidence of gingival hypertrophy.

**Supplementary Information:**

The online version contains supplementary material available at 10.1007/s10103-025-04444-5.

## Introduction

During orthodontic treatment, forces are used to move the teeth, which leads to a physiological reaction in the surrounding tissue. This process requires remodeling of the alveolar bone and the periodontal ligament (PDL), with bone resorption at pressure sites and bone formation at tension sites, depending on the force applied and the reaction of the PDL [1]. Accelerating orthodontic therapy can reduce long-term risks such as caries and gingivitis [2–4].

Pain, a common side effect of orthodontic tooth movement (OTM), is associated with local inflammation [5, 6]. It begins within two hours of force application, peaks between 12 and 24 h after application, and decreases to an almost imperceptible level by the seventh day [5, 7, 8].

In addition, the elastics as part of fixed orthodontic appliances (FOAs) promote bacterial growth and plaque formation, which leads to gingival inflammation [9–11]. This inflammation can cause gingival hypertrophy due to the increased extracellular matrix. Poor oral hygiene exacerbates the build-up of plaque and the accumulation of bacterial colonies, especially around orthodontic brackets [9, 10].

Photobiomodulation (PBM) that is also known as low-level light therapy (LLLT) is a therapeutic method that uses light (the source is laser or light emiting diodes-LED) to stimulate tissue regeneration, reduce inflammation and relieve pain [12]. The effect is based on the absorption of light in the mitochondria, which stimulates ATP production through oxidative metabolism. The source suggests that the mechanisms of PBM have evolved to consider not just ATP production but also broader cellular targets. This includes ion channels (e.g. opsins), the cytoskeleton, and bio photonic signaling pathways [9, 10, 13, 14]. PBM generally uses light with an irradiance of 5 mW/cm² to 500 mW/cm² and wavelengths of 600 to 1000 nm (red to near infrared) [15–17]. It uses low power light to obtain physiological response without thermal or cytotoxic effects [18]. Light therapy has been a subject of investigation across various fields for many years. Despite some differences in the light sources, both low-level lasers and light-emitting diodes (LEDs) have demonstrated therapeutic effects due to similar mechanism of action [19–22]. Despite differences, LED showed to be effective in many treatments, however their effects may be influenced by the setting of the study (in vitro, animal, clinical) [19, 20]. Both methods have been found to promote healing, reduce inflammation, and enhance cell function, but the choice between using a laser or LED may depend on the treatment goals, anatomical area, available evidence, availability, and specific patient needs [19]. Compared to lasers, LEDs are generally cheaper and easier to use [23].

Photobiomodulation (PBM) is recognized as a safe and effective treatment method. When the appropriate irradiation regimen is followed, no adverse side effects have been reported for PBM on the irradiated tissue in the head and neck region [16, 24, 25].

The main benefits of PBM in orthodontics could include pain relief and acceleration of tooth movement, which could shorten the overall duration of treatment [24, 26–29].

Since OTM depends on alveolar bone remodeling, it has been demonstrated that the rate of orthodontic tooth movement is related to treatment time and faster bone remodeling leads to increased OTM [30]. In the meta-analysis by Yavagal et al. an acceleration of OTM by PBM was observed in 5 of 8 included studies, while a later meta-analysis by Olmedo-Hernandez concluded that the evidence was insufficient to draw conclusions at the current state of knowledge [31]. Whilst there is increasing evidence of beneficial effects of laser PBM in patients undergoing OTM, there is currently much less certainty about the efficacy of LED PBM [32]. Farhadian et al. compared placebo, 810-nm laser and 640-nm LEDs during orthodontic canine retraction and reported a significant 60% acceleration of OTM rate in the laser group compared to placebo, while the acceleration with LED PBM was lower at 26% and not statistically significant; in contrast, Guray et al. reported a significant acceleration of OTM stimulated by 850-nm LEDs compared to placebo. The meta-analysis by Bahrami et al. reported a combined acceleration with LED-PBM of 33% compared to placebo [23, 33, 34].

Previously, studies in human participants undergoing OTM have shown altered levels of markers of bone remodeling such as osteoprotegerin, interleukin-1β and receptor activator of nuclear factor-kappa Beta ligand (RANKL), indicating an increased rate of bone metabolism [4]. The results of the meta-analysis show that LED PBM can induce changes in bone metabolism which could lead to an acceleration of OTM [32, 35–37]. However, there are many inconsistencies and the lack of clinical evidence limits a more accurate assessment, so more randomized trials under different clinical conditions are needed, especially in regards to effects of LED in patients undergoing OTM [35–37]. Furthermore, to our knowledge, PBM has not yet been investigated whether it can reduce gingival hypertrophy during OTM.

The aim of our clinical study is to clarify the role of irradiation of dental and periodontal tissues with a PBM from light-emitting diode (LED), whether it affects the speed of tooth movement, periodontal tissue inflammation and subjective pain perception during orthodontic tooth movement in the initial levelling phase with FOA.

## Materials and methods

The study was approved by the National Medical Ethics Committee of the Republic of Slovenia for under reference number 0120–566/2021/6 and was conducted from March 2022 to July 2022. All participants and, in the case of minors, their parents or legal guardians signed a consent form.

### Participants

We initially enrolled 35 participants in this prospective, randomized clinical trial. The inclusion criteria were as follows: Patients were between 13 and 19 years old, had permanent dentition, were non-smokers, were expecting orthodontic treatment with FOA due to moderate crowding in the anterior sector and were willing to fully participate in the study and attend all required sessions. The exclusion criteria were; temporomandibular joint pathology, treatment or conditions that could interfere with bone remodeling. Patients with fillings, fixed prosthetic restorations or those who had previously undergone endodontic treatment were also excluded from the study to avoid potential bias [38].

Participants were randomized into two groups: the experimental group, which was exposed to PBM with LED, and the control group, which was exposed to a non-therapeutic placebo light using a standard low-power incandescent lamp that was visually indistinguishable from the therapeutic light. Randomization was performed sequentially when participants entered the study.

All patients were asked about their general and dental health with the FDI health questionnaire. A comprehensive dental examination was performed according to the documented protocol. Participants were examined before FOA placement and then 1 and 4 weeks after placement. Placement of the FOA and application of orthodontic forces were performed by either a specialist or an orthodontic resident, adhering to professional standards by attaching orthodontic brackets to the teeth and inserting a 0.014-inch diameter round wire made of a super elastic nickel-titanium alloy. After the 4-week study period, participants were informed of their measurement results and participants in the control group were offered therapeutic light treatment (PBM with LED). Throughout the study, all participants were regularly encouraged to maintain and improve their oral hygiene.

### Sample size calculation

The sample size was calculated for the difference between experimental and control group in OTM rate and gingival hypertrophy. A test power of 0.80 and a statistical significance level of 0.05 (alpha) were assumed. A sample size calculation tool for t-test was used to determine the sample size. In order to differentiate the groups in OTM rate inclusion of at least 14 participants would be required. In order to differentiate the groups in terms gingival, hypertrophy at least 31 participants would have to be included.

### PBM protocol

The experimental group received PBM with LED light from the Ortholumm ML5/1 therapeutic device, which performed a “sweep” pulsing pattern from 50 to 2000 Hz (Voltan d.o.o., Slovenia). The therapeutic consisted of three types of LED and emitted light of wavelengths of 625 nm, 660 nm and 850 nm simultaneously with an average irradiance of 16 mW/cm². The power of the individual wavelengths was distributed unevenly: 625 nm contributed 24%, 660 nm contributed 71% and 850 nm contributed 5% of the total illumination power. The irradiation emitted per minute was 0.960 J/cm².

The PBM light was administered extra-orally to irradiate the lips, buccal mucosa, teeth and periodontal tissue. During PBM therapy, the subjects kept their teeth in occlusion, supported by a plastic lip retractor which held the lips and cheeks in an open position. The therapeutic device was positioned about 5 cm from the mouth. Once the device was correctly aligned, the light source was activated. Light therapy was administered twice a week for 4 consecutive weeks, with each session lasting 10 min and a minimum interval of 2 days between sessions. This protocol was similar to that recommended by Impellizzeri et al. which included 4 treatments within the first 14 days [3]. The total irradiation time during the study was 80 min. The first therapy session was performed within two days of FOA placement and activation. On the measurement days, light therapy was only administered after all measurements had been completed.

The control group was exposed to a non-therapeutic light from a standard low-wattage incandescent light bulb, using the same protocol as the experimental group placebo device was visually indistinguishable from the therapeutic light. Neither the therapeutic nor the placebo light emitted ultraviolet light. Participants wore protective goggles during each irradiation session.

### Recorded variables

To evaluate the effect of light therapy, we measured the following parameters in both the experimental and control (placebo) groups:

- The extent of OTM during treatment, determined by measurements on 3D models obtained by intraoral scanning of the dental arches,
- Plaque index (PI) and sulcus bleeding index (SBI) as well as the assessment of gingival hypertrophy around the teeth with orthodontic brackets,
- The patients’ subjective perception of pain using the visual analogue scale (VAS).

The iTero Element Scanner (Align Technology Inc., USA) was used to accurately record the positions of the teeth. This included scanning the upper dental arch, the lower dental arch and their relationship to each other. In the mandible, the scan included the entire alveolar ridge together with the teeth. In the maxilla, the scan also included the entire hard palate. According to our protocol, the scans were performed at three key time points: first before FOA insertion, then 1 week after insertion and finally 4 weeks after insertion.

MeshLab 2022.02 software (Consiglio Nazionale delle Ricerche and Istituto di Scienza e Tecnologie dell’Informazione, Italy) was used to analyse the virtual 3D study models. The models were compared 1 week and 4 weeks after placement with the initial state before FOA placement.

**Fig. 1:**
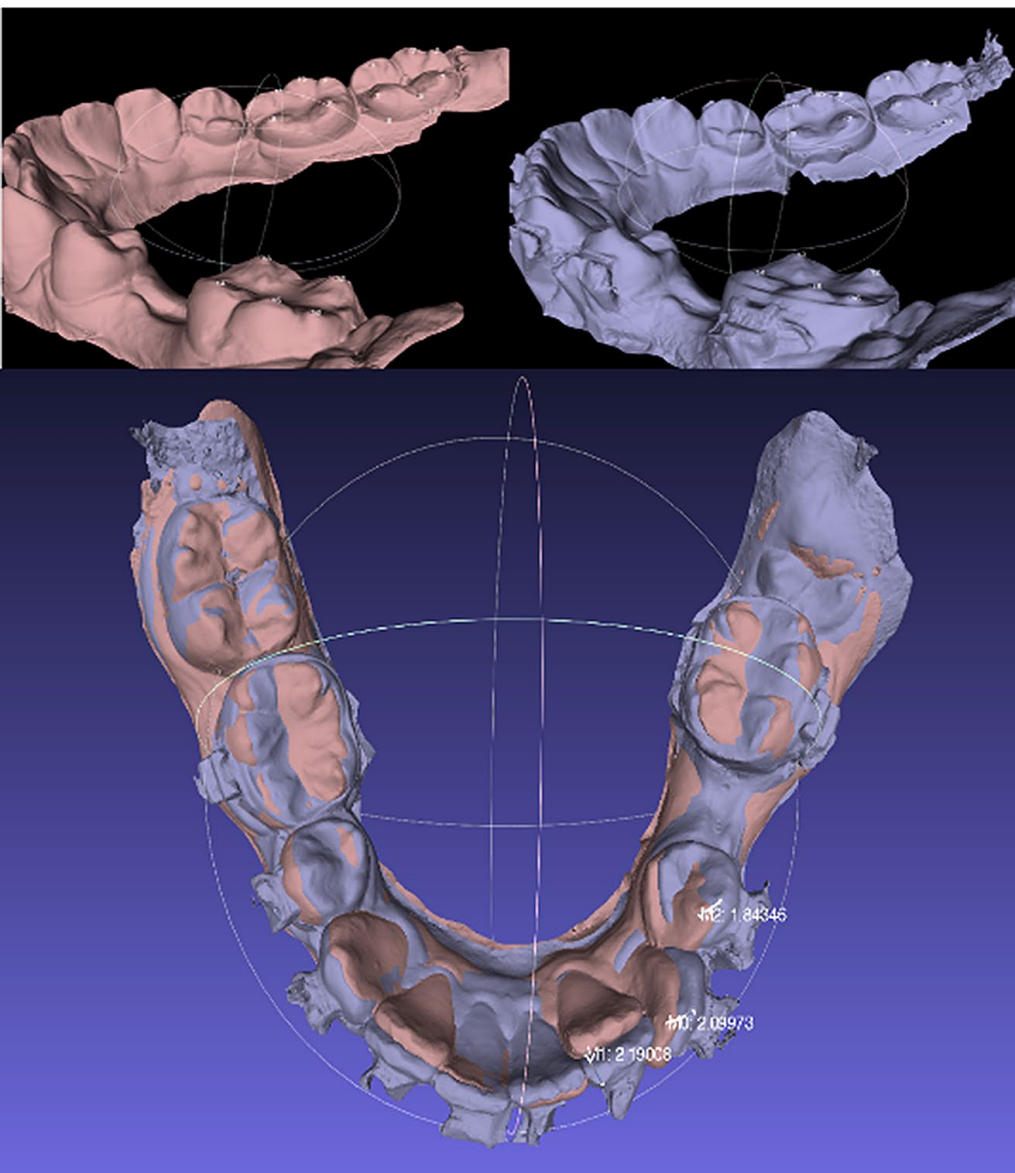
**A** (upper): Determining points on molars for model registration in *MeshLab software*. **B** (lower): Measurement of tooth movements using *MeshLab software*

For model registration, FOA point-based best-fit method was used to minimize the average distance between the corresponding points in the models. Initially, both models of the same jaw were registered using the “point-based glueing” function. In the mandible, identical reference points were identified and marked on both models, strategically chosen on the convexities of the molars and on the lingual gingival plane of teeth that were not involved in the FOA or were expected to experience minimal movement (Fig. 1A). In the maxilla, points were primarily selected on the 2nd and 3rd palatal rugae, as previous studies have shown that the morphology of this region is only minimally altered during orthodontic procedures [39, 40]. On average, 10 points on each model were selected for registration. After registration of the digital models, the tooth movements were evaluated. The displacement between two fixed reference points at the incisal edge (for incisors and canines) or at the convexity of the occlusal surface (for premolars) was measured, as shown in Fig. 1B.

To evaluate gingival tissue inflammation, the plaque index (PI) and sulcus bleeding index (SBI) were determined and gingival hypertrophy (GH) around teeth with orthodontic brackets was assessed. When evaluating the PI, we used a periodontal probe to assess the presence of plaque along the tooth-gum interface. When assessing the SBI, any occurrence of sulcular hemorrhage was observed after probing with a periodontal probe along the tooth-gum junction. Four specific sites around each tooth were assessed: mesial, central buccal, distal and central lingual. A dichotomous scoring system was used in the assessment, where a score of 0 indicated the absence of plaque/bleeding at the site, while a score of 1 indicated the presence of plaque/bleeding. The PI and SBI were then calculated and expressed as a percentage (%). Patients were instructed to brush their teeth thoroughly before the examination. Following the examination, patients were given comprehensive education on oral hygiene and the importance of plaque removal in maintaining oral health.

**Fig. 2:**
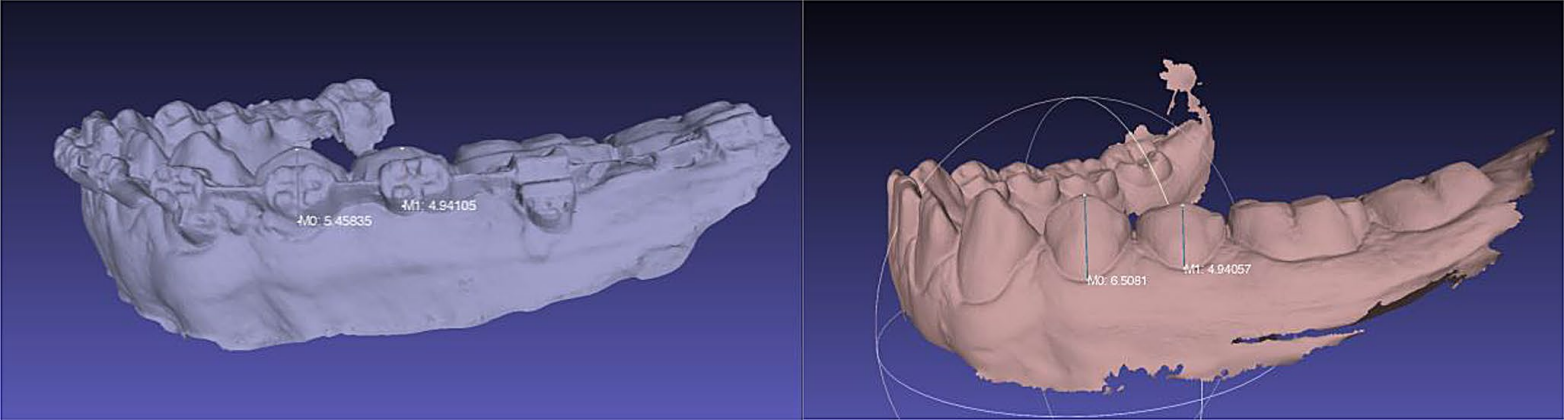
Evaluation of gingival hypertrophy on the digital study model. The right lower arch represents the state before FOA placement and the left 4 weeks post-placement. Tooth 34 has a score of 1 (hypertrophy present) and tooth 35 has a score of 0 (no hypertrophy present)

Gingival hypertrophy (GH) was assessed before placement of the FOA and 4 weeks after placement. GH was recorded where the gingival tissue extended beyond the enamel-cement junction on all teeth that had orthodontic appliance on their surface. The measurements were recorded dichotomously: A score of 0 was assigned if the gingival tissue was at the level of the enamel-cement junction, and a score of 1 was assigned if the gingival tissue extended at least 1 mm beyond the enamel-cement junction. The clinical measurements were verified by comparing scanned dental arches. In the digital model, the GH was measured from the center of the incisal edge of the tooth perpendicular to the gingival margin on that tooth, as shown in Fig. 2.

The subjective perception of pain was assessed using a questionnaire based on the visual analogue scale (VAS), a widely used method of pain assessment [41]. Patients were instructed to mark the intensity of their pain on a 10 cm horizontal line, with the endpoints labelled “I feel no pain at all” and “The pain is unbearable”. Pain intensity was recorded at three time points: before FOA placement, 1 week and 4 weeks after placement. The VAS was used to assess pain intensity both at rest and during chewing. For the analysis, the distance from the beginning of the line to the patient’s marker was measured and converted into a numerical pain score between 0 and 10.

### Statistical analysis

The data were analyzed using the SigmaPlot statistical programme (Sigmaplot 14.0 Systat, San Jose, USA). Statistically significant differences between the groups were determined with non-parametric and parametric tests. The normality of the sampling distribution was tested using the Shapiro-Wilk test. If the p-value was less than 0.05, non-parametric methods were used for the testing of variables.

Before the start of treatment, the therapeutic (PBM) and control (placebo) groups were compared by age with the t-test, by gender with the Fischer exact test and by tooth category (incisor, canine, molar) with the chi-square test. Differences between the PBM and placebo groups at different time points were tested using the t-test for independent samples. The Mann-Whitney test was used to assess the differences between the groups in terms of PI, SBI, GH and pain perception according to the VAS and OTM.

## Results

The study was conducted on 35 participants who underwent initial measurements and were randomly assigned to the experimental (PBM) or the control (placebo) group. Data obtained from 32 participants were analyzed, with 18 individuals in the control group and 14 in the experimental group. Three patients from the experimental group were excluded due to irregular attendance at measurements and light therapy sessions. The average age of the participants was 14.6 ± 2.0 years.

Table 1 presents the distribution of participants by gender and age in the experimental (PBM) and control (Placebo) groups. Non-parametric statistical tests indicated no significant differences in age between the PBM and Placebo groups (Mann-Whitney U test, *p* = 0.571), nor in gender distribution (Fisher’s exact test, *p* = 0.446).

**Table 1:** The distribution of participants by gender and age in the experimental (PBM) and control (Placebo) groups

		all	PBM	Placebo
Number of patients, N (%)		32 (100%)	14 (43.75%)	18 (56.25%)
Gender N (%)	male	10 (31.25%)	3 (21.43%)	7 (38.89%)
	female	22 (68.75%)	11 (78.57%)	11 (61.11%)
Age [years]	median	14.0	13.5	15.0
	interquartile range	13.0–16.0	13.0–16.5	13.0–16.0
	span	12–19	12–19	12–18

**Fig. 3:**
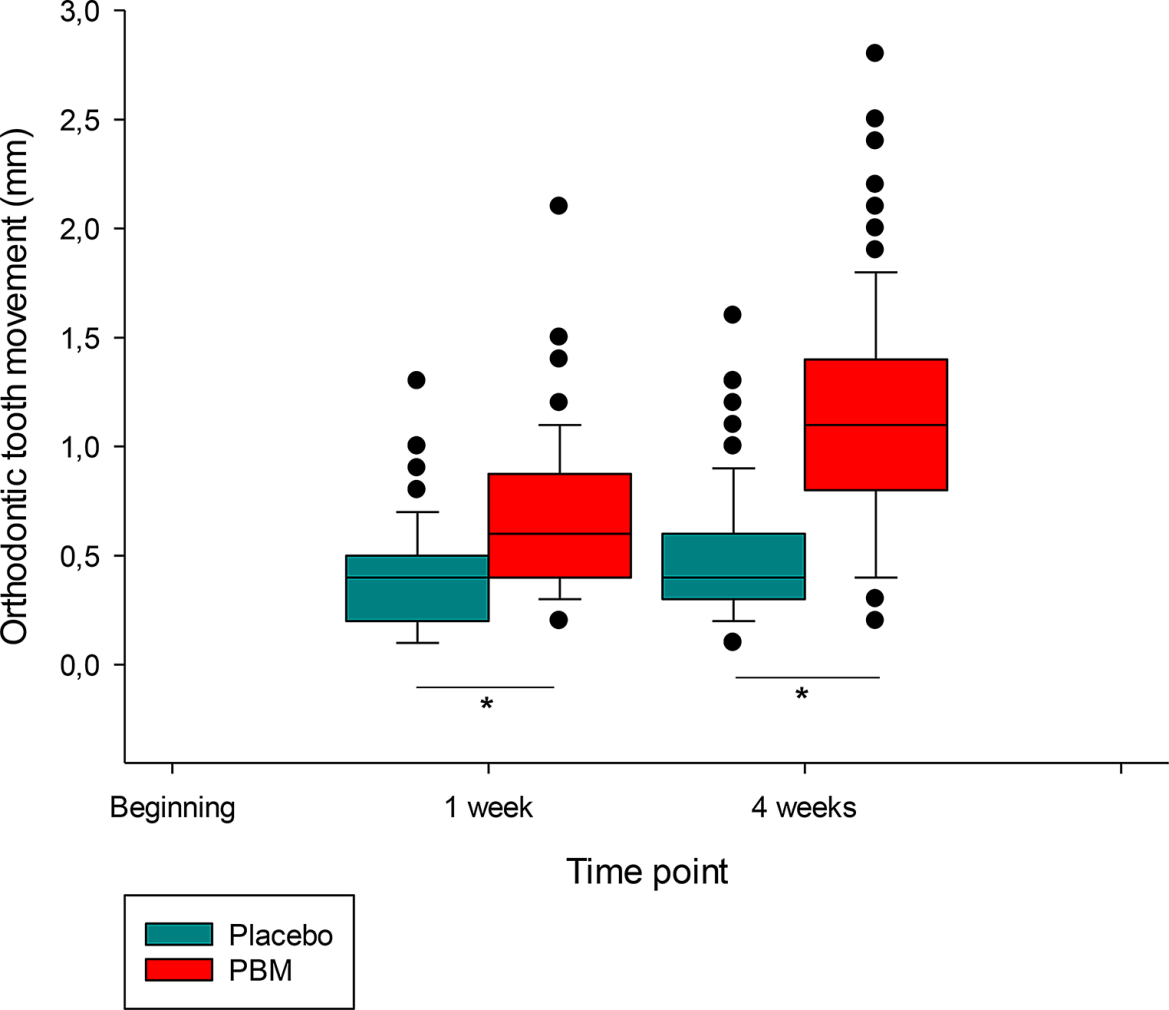
Tooth movement after 1 and 4 weeks in the experimental (PBM) and control (placebo) group. *marks a statistically significant difference (*p* < 0.05)

In the PBM group exposed to therapeutic light, OTM was statistically significantly greater at 1 week and 4 weeks post-placement of FOA, compared to the baseline measurements (Mann-Whitney U test, *p* < 0.001), as shown in Fig. 3. At the 1-week mark, the median OTM in the experimental group was 0.1 mm greater than the baseline. At the 4-week mark, the median OTM in the experimental group was 0.5 mm greater than the baseline.

There were no statistically significant differences found in the PI or SBI between the PBM and placebo groups. Using the Mann-Whitney U test, no statistically significant differences were found between the groups at any time points (*p* > 0.050). Table 2 presents the median values and interquartile range of the PI and SBI in both the PBM and placebo groups.

**Table 2:** PI and SBI before placement (0), 1-week post-placement (1) and 4-weeks post-placement (2) of FOA in PBM and placebo group

		PBM	Placebo	p-value
PI 0 [%]	median	20.98	14.28	/
	interquartile range	13.78–30.58	8.92–31.31	
PI 1 [%]	median	14.51	14.45	0.917
	interquartile range	8.93–29.02	10.04–26.34	
PI 2 [%]	median	20.54	12.75	0.080
	interquartile range	11.82–26.34	4.01–19.27	
SBI 0 [%]	median	2.24	0.00	/
	interquartile range	0.00–4.24	0.00–2.45	
SBI 1 [%]	median	3.64	2.64	0.817
	interquartile range	0.00–7.96	0.22–5.32	
SBI 2 [%]	median	3.57	2.44	0.751
	interquartile range	0.06–5.81	0.00–5.58	

**Fig. 4:**
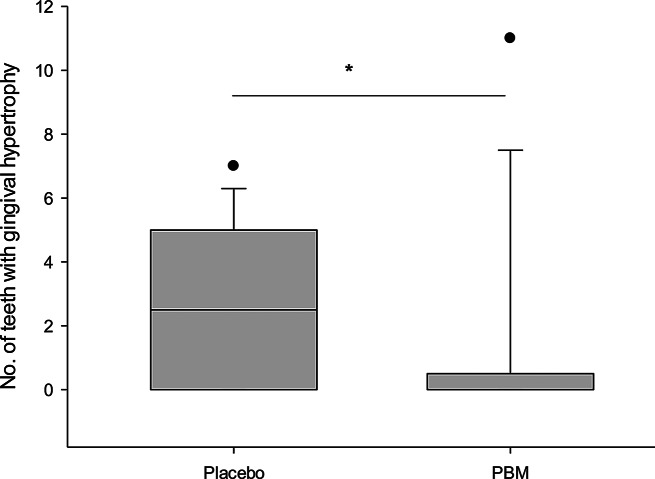
Incidence of gingival hypertrophy after 4 weeks of FOA treatment. *marks a statistically significant difference (*p* < 0.05)

GH was observed in 21.4% of subjects in the PBM group and 55.6% in the placebo group. A statistically significant difference in the incidence of GH was observed between the PBM and placebo groups after 4 weeks of FOA placement (Mann-Whitney U test, Using the Mann-Whitney U test (*p* > 0.05). Figure 4 presents incidence of teeth with gingival hypertrophy after 4-weeks of treatment with FOA.

**Fig. 5:**
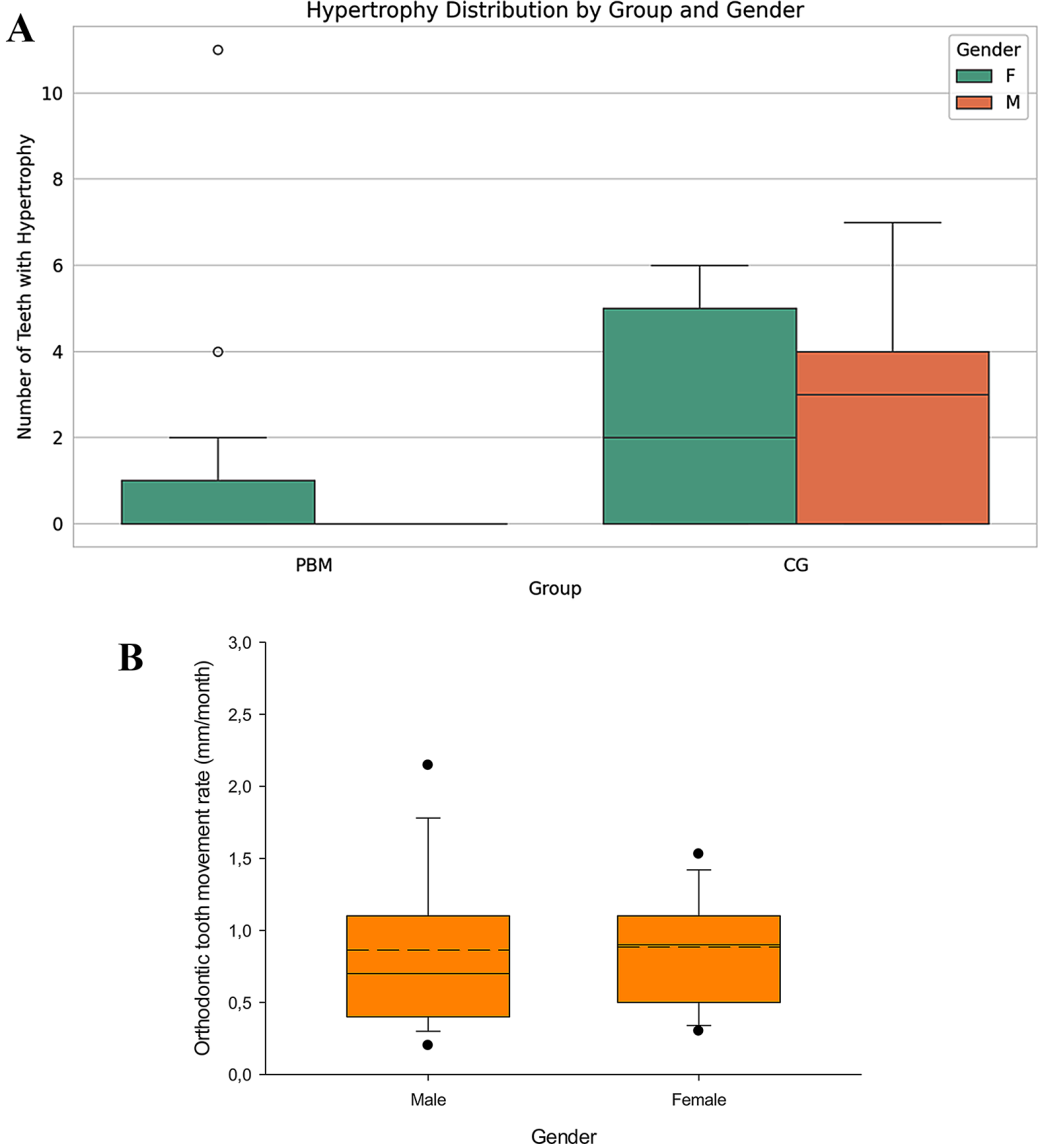
Comparison of incidence of gingival hypertrophy (**a**) and monthly OTM rate (**b**) between boys and girls (combined sample of both research groups)

Since a possible early onset of adolescence in girls and the subsequent peak of steroid hormones could influence the tendency of girls and boys to hypertrophy and the rate of OTM, children were compared by gender. There was no significant difference in gingival hypertrophy (*p* = 0.858) or OTM rate after one month of treatment (*p* = 0.560) (Fig. 5).

Partial Least Squares (PLS) regression analysis using gender and group as predictors for hypertrophy count was performed. The analysis showed R-squared of 0.068, predicting 6.8% of variance in gingival hypertrophy. Neither treatment group nor gender showed statistically significant values (Table 3).

**Table 3:** PLS regression multivariate analysis. R-square = 0.068

Variable	Coefficient	CI_Lower	CI_Upper	*p*-value
Group_PBM	-0.749	-1.585	0.305	0.178
Gender_male	-0.273	-1.043	0.580	0.536

**Fig. 6:**
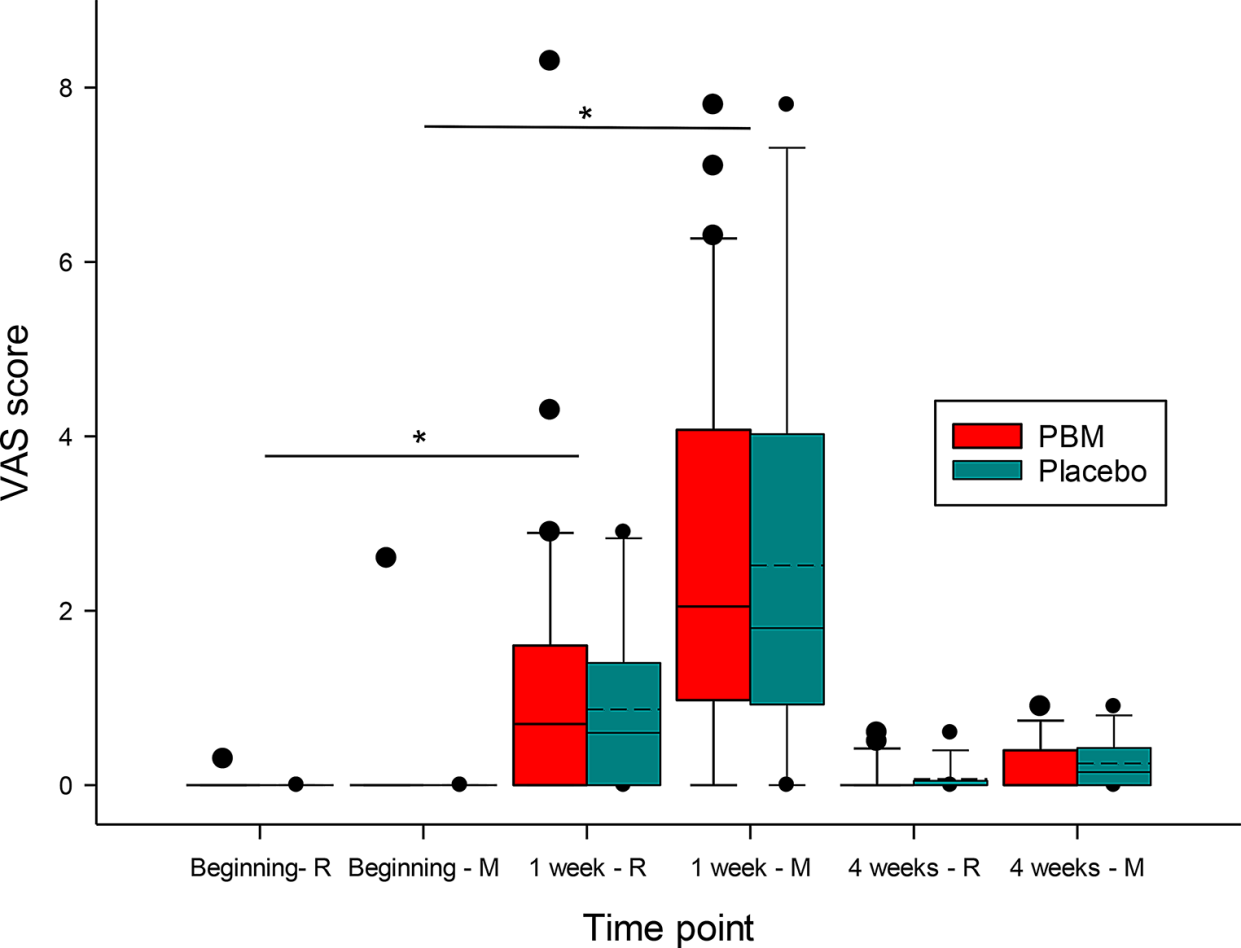
Level of pain perception at rest (R) and during mastication (M). *marks a statistically significant difference (*p* < 0.05)

There were no statistically significant differences in subjective pain perception at any time point between the PBM and placebo groups. Figure 6 illustrates the level of pain perception at rest (R) and during mastication (M).

A comparison between VAS 0 and VAS 1 revealed that subjective pain perception after 1 week, both at rest and during mastication, was statistically significantly higher (*p* < 0.05) than before the placement of FOA (Table 4). These results were consistent within both the PBM and placebo groups, respectively. However, when comparing VAS 0 and VAS 2, no statistically significant differences were found in either group (*p* > 0.05).

**Table 4:** The subjective perception of pain according to the VAS from 0 to 10 at rest and during mastication in a sample of all subjects

	Rest	Mastication
VAS 0	0.0 ± 0.1	0.1 ± 0.5
VAS 1	1.2 ± 1.7	2.6 ± 2. 2
VAS 2	0.1 ± 0.2	0.2 ± 0.3

## Discussion

The present study shows that PBM using LED light significantly accelerates OTM both 1 and 4 weeks after the onset of FOA treatment. In addition, PBM therapy had a positive effect on periodontal tissue, as evidenced by a lower incidence of gingival hypertrophy in the PBM group. In contrast, no statistically significant differences in plaque accumulation or sulcus bleeding were observed between the experimental and control groups. Furthermore, PBM did not significantly reduce the subjective pain perception of the participants during the levelling phase of orthodontic treatment.

We observed a significant difference in the amount of tooth movement between the PBM and placebo groups. After 1 week of FOA treatment, there was a median movement of 0.5 mm OTM in the PBM group, while there was a movement of 0.4 mm OTM in the placebo group. Although the difference was statistically significant, this small difference of 1 week may not be clinically relevant. The observed movement in this initial periodontal phase of OTM is mainly due to the compressibility of the periodontal ligament [42, 43]. In contrast to early OTM, the difference in the extent of OTM after 1 month of treatment was both statistically and clinically significant (the median extent of OTM in the PBM group was 1.1 mm compared to 0.6 mm in the placebo group). We can conclude that only a minimal amount of OTM occurred in the placebo group. This can be attributed to the second, “lag” phase of OTM and suggests that PBM significantly shortened the lag phase in treated patients. Whether shortened lag phase of OTM might affect root resorption would need to be further investigated [44]. As the early phases of OTM have the greatest impact on pulpal blood flow, in future studies it would be worth investigating whether PBM could positively influence the condition of the dental pulp in the early phases, which are considered to be the most risky from the pulpal perfusion perspective [45]; and whether that would later result in altered pulpal neural sensitivity [46]. In a similar study, Impellizzeri et al. observed an average tooth movement of 1.98 mm in the PBM group and 1.35 mm in the placebo group after one month. It is noteworthy that only premolar movements were measured in this study [3]. Meta-analysis reported of Grajales et al. reported overall around 0.5 mm increase of OTM per month in PBM groups, which is consistent with our results [35]. Al Shahrani et al. similarly reported faster tooth movement in subjects exposed to PBM than in the placebo group in a systematic literature review [24]. Recent review reported that increased rate of OTM did result in shorter treatment time [47].

Most of the evidence for the use of PBM in orthodontic treatment comes from studies using lasers [48]. Red (660 nm) and near-infrared (810/980 nm) lasers have been shown to induce proliferation of human mesenchymal stem cells in vitro [49]. In orthodontic retraction of canines, Farhadian et al. observed a higher rate of orthodontic retraction of canines in the laser group (60% increase) compared to the LED group, which observed a 26% increase compared to untreated controls [33]. It should be noted that the laser group was irradiated with a higher irradiance compared to the LED group.

A notable difference between LEDs and lasers is that the coherent nature of laser light — where all photons are in phase — can possibly allow deeper penetration into tissue. LEDs, on the other hand, emit incoherent light, usually in a more divergent beam compared to lasers, which can lead to different surface reflection and scattering pattern, possibly contributing to clinical result measured empirically. Due to their properties, LEDs are considered suitable for treating superficial tissues. Studies emphasise their flexibility of use and cost efficiency [23]. In our study, LED therapy was applied directly to the teeth and gingiva did show effect on the treated the target tissue.

In a comprehensive review of the literature, we found that authors often reported tooth movement in millimeters without specifying the exact time frame in which the movement occurred. This lack of standardization therefore poses a challenge when comparing results.

To quantify OTM in the lower dental arch, we used the best-fit method to register 3D models. This technique is currently the most reliable approach for registration in the mandibular region, as there are no stable anatomical landmarks on 3D models of the mandible that remain completely unchanged during orthodontic treatment. To address this issue, we have refined this method by including multiple reference points on teeth that are not affected by the FOA, minimizing positional changes when models are superimposed. When registering the maxillary arch, we mainly relied on the third palatal mucosa and its dorsal region. This region has demonstrated exceptional anatomical stability and is considered the most accurate method for superimposing 3D models of the maxilla [39, 50].

Although studies suggest that PBM may affect the amount of PI in mucositis patients, the results of our study show no statistically significant differences between the PBM and placebo groups in terms of PI measurements [51]. This result was to be expected as our study design involved random allocation of subjects, regardless of their oral hygiene practices or attitudes.

We observed no statistically significant differences between the groups when assessing the SBI. FOA provides a substantial retention surface for plaque accumulation by increasing the likelihood of impaired oral hygiene and consequent gingivitis. Stain et al. reported that PBM therapy accelerated the resolution of gingivitis in patients after FOA removal [11]. However, we found no statistically significant difference in SBI between subjects who received therapeutic or non-therapeutic light therapy during the first month of orthodontic treatment with FOA.

We investigated the occurrence of developed gingival hypertrophy in patients after 4 weeks of orthodontic treatment with FOA, as this manifestation typically occurs only after a longer period of time. The incidence of hypertrophy was significantly higher in individuals in the placebo group than in the PBM group. In particular, hypertrophy was observed in 55.6% of subjects in the placebo group. This result is in close agreement with the findings of Vincent-Bugnas et al. who found a prevalence of hypertrophy in 49.7% of subjects undergoing orthodontic therapy with FOA in a larger cohort of 193 subjects [10]. In our clinical study, PBM was observed to contribute to a significant reduction in gingival hypertrophy. This effect is probably due to the anti-inflammatory and anti-proliferative properties of PBMT on periodontal tissue, which help to control and reduce the overgrowth of gingival tissue caused by chronic localised inflammation [52, 53].

It is unlikely that the observed differences are due to poorer dental cleaning practices of the subjects in the placebo group, as shown by the lack of a statistically significant difference in PI between the PBMT and placebo groups. It is noteworthy that a higher PI was found in the PBM group. In addition to oral hygiene, numerous other factors can influence the occurrence of gingival hypertrophy independent of plaque control. These factors include direct mechanical irritation of the gingiva, oral respiration, immune responses to metal and non-metal ions, syndromic diseases, vitamin C deficiency, chronic inflammation, hormonal fluctuations and medication effects [10, 54, 55]. Medications that contribute to gingival overgrowth include anticonvulsants, immunosuppressants and calcium channel blockers [56].

Vincent-Bugnas et al. reported that the composition of plaque exerts a stronger influence on gingival hypertrophy than its sheer quantity [10]. Given the reduced incidence of gingival hypertrophy in the experimental group, we hypothesize that PBM therapy might influence the characteristics or composition of the plaque biofilm. This hypothesis would be worth pursuing in later trials.

No statistically significant differences were found between the PBM and placebo groups when patients’ pain perception was assessed during the first month of orthodontic treatment, neither after 1 week nor after 4 weeks. In particular, no differences were found in the perception of pain at rest or during chewing. Consequently, we cannot claim that PBM significantly alleviated pain perception in the subjects in the experimental group during active orthodontic treatment. It is noteworthy that this result differs from certain other studies [57, 58].

In the study conducted by Sfondrini et al., they reported the efficacy of PBM in relieving pain intensity within the first 12 h after orthodontic force application. Specifically, they administered PBM with an energy density of 7.5 J/cm2 [5]. Similarly, a systematic review by Zhi et al. concluded that lasers with an energy density of 0 to 10 J/cm2 effectively reduced the perception of pain 1, 2 and 3 days after the application of orthodontic forces [58].

In contrast, PBM with a lower energy density of 1.0 J/cm2 was used in our study, which may be the reason for the lack of recognizable differences in pain perception. In addition, a third independent group included in later studies, consisting of subjects treated without additional therapy (such as a placebo), would provide valuable comparative insights.

Bezerra et al. used near infrared LED PBM after insertion of orthodontic elastomeric separators. They observed significant reduction in pain levels in participants. They observed significant differences in pain levels immediately and 48 h after insertion of elastomeric separators. After 6 days they no longer observed any difference between groups which is similar to our finding [59].

We then assessed VAS measurements in all subjects as a function of time since FOA placement. Our results showed a statistically significant increase in subjective pain perception after 1 week compared to baseline measurements. Conversely, we found no statistical difference in the subjects’ pain scores over time between baseline measurements before FOA and measurements after 4 weeks of therapy. This trend persisted both at rest and during chewing.

Hussain et al. point out that pain begins approximately 4 h after orthodontic force insertion and reaches its peak intensity 24 h after insertion before gradually subsiding within 7 days [60]. Similarly, in a study on pain perception during orthodontic separator insertion conducted on a sample comparable to ours, Bergius et al. reported that the maximum pain intensity was experienced at the 24-hour mark. Their results showed that 42% of the subjects still experienced pain on the 7th day after insertion [7].

We documented the first perception of pain 1 week after FOA placement, which is consistent with the time course observed in previous studies. Our results confirm that pain perception remains statistically significantly elevated at day 7 after placement. To address this limitation and increase the completeness of our measurements, future studies could include an evaluation of VAS scores at the 24-hour mark after implantation to capture the maximum pain intensity.

## Conclusion

Significantly accelerated tooth movement was observed in the PBM group both 1 week and 4 weeks after FOA placement. In addition, PBM had a positive effect on periodontal tissue, as evidenced by a lower incidence of gingival hypertrophy in patients undergoing PBM therapy. In contrast, no statistically significant differences in plaque volume or sulcus bleeding were observed between the PBM and placebo groups.

In our study, we could not confirm the hypothesis that patients who received PBM therapy would have a lower pain sensation compared to the placebo group. Consequently, we cannot confirm that PBM significantly reduces the perception of pain in individuals within the PBM group undergoing the levelling phase of active orthodontic treatment.

## Electronic supplementary material

Below is the link to the electronic supplementary material.

Supplementary Material 1

## Data Availability

No datasets were generated or analysed during the current study.
