# Supplementary material for: Effect of led photobiomodulation on tooth movement, gingival hypertrophy and pain in response to treatment with fixed orthodontic appliance
Source: Lasers Med Sci. 2025 Apr 18;40(1):200. doi: 10.1007/s10103-025-04444-5 (PMC12008064; doi:10.1007/s10103-025-04444-5)
Supplement: Supplementary file 1 — Supplementary Material 1 [file 10103_2025_4444_MOESM1_ESM.doc]

Assessed for eligibility

(n = 60)

**Enrollment**

Excluded (n = 24)

Not meeting inclusion criteria

(n = 2)

Refused to participate

(n = 23)

Randomized (n = 35)

#

**Allocation**

**Follow up**

**Analysis**

Allocated to PBM

(n = 17)

Received allocated intervention (n = 14)

Allocated to placebo

(n = 18)

Received allocated intervention (n = 18)

Lost to follow up

(n = 3)

(participation was too time consuming, had too many school chores)

Lost to follow up

(n = 0)

Analyzed (n = 14)

Excluded from analysis

(n = 3) (lost to follow up)

Analyzed (n = 18)

Excluded from analysis

(n = 0)
